# Health extension service utilization barriers in East Gojjam Zone, Northwest Ethiopia: qualitative study

**DOI:** 10.11604/pamj.2021.40.150.31287

**Published:** 2021-11-11

**Authors:** Bewket Yeserah Aynalem, Misganaw Fikrie Melesse

**Affiliations:** 1 Department of Midwifery, Debre Markos University, Debre Markos, Ethiopia

**Keywords:** Ethiopia, health services, delivery of health care, urban population, rural population, communicable diseases, environmental health, food inspection, qualitative research

## Abstract

**Introduction:**

the health extension service is a package that aims to improve primary healthcare services, mainly in rural areas through an innovative community-based approach that focuses on prevention, healthy living, and basic curative care which is implemented by the health extension workers using the health post as a center of care. Thus, this study aimed to explore the barriers of health extension service utilization in East Gojjam Zone, North West, Ethiopia.

**Methods:**

qualitative study was conducted from Feb 16 to May 30, 2021, on the barriers to health extension service utilization among households in East Gojjam Zone. The data was collected with focus group discussion and in-depth interviews to address our objective. Study participants were selected purposively until the required data was saturated. The data was analyzed under selected themes based on the guide and summarized manually.

**Results:**

respondents reported that there were lots of reasons that preclude proper utilization of the health extension packages both in urban and rural households in East Gojjam Zone-like negligence, previous experience, misinterpretation of the health extension packages, the religion they believe, unavailability of water continuously, wrongly utilization of the packages rather than their purpose and the environment they live.

**Conclusion:**

there are still lots of barriers regarding health extension package utilization in the East Gojjam Zone. Working on households on purpose and utilization of health extension packages in a religiously and culturally acceptable manner is advisable. Further research on reported barriers is needed.

## Introduction

Health extension package (HEP) is an essential health intervention that has the aim of prevention and increment of community-health awareness targeting households particularly women/mothers at the kebele level [1-5]. In Ethiopia, it was launched by the government in 2003 by training new health care, Health Extension Workers (HEWs) using health posts(HP) as a center of services especially for the rural community [6]. The Ethiopian federal ministry of health (FMoH) annual report indicated that 60-80% of mortality is due to preventable communicable diseases like Tuberculosis (TB), Human Immune Deficiency Virus/Acquired Immunodeficiency Syndrome (HIV/AIDS), pneumonia, and malaria [7]. The distribution of these diseases is mainly due to poor socioeconomic status, low level of awareness about health, and inadequate health service delivery across the country [8]. the mortality due to communicable diseases is huge but could be prevented through appropriate utilization of the health extension service packages [9].

In Ethiopia, stunting due to malnutrition (38%) of under-five children is a serious problem. Amhara region accounts for the highest proportion (46%) particularly East Gojjam Zone [10]. Ethiopia has fully launched and addressed the HEPs all over the country but the utilization is still low like treated and safely stored water use (8.5%), improved toilet use (6%), handwashing with soap practice (7%), under5 mortality (30%) and unmet need for family planning is 22% [10, 11]. This showed that a large segment of the population in Ethiopia is still low in implementing the health extension packages. Therefore, the study aimed to explore the barriers of health extension service utilization in the East Gojjam Zone.

## Methods

**Study setting and population:** this study was conducted in East Gojjam Zone households, Amhara region, Northwest Ethiopia from February 16 to May 30, 2021. East Gojjam zone is one of the Amhara regional state zones of Ethiopia with a capital city of Debre Markos town (located at 300 km from Addis Ababa, the capital city of Ethiopia, and 265 km from Bihar Dar, the capital city of Amhara). As the zonal health department report indicated that East Gojjam zone has a total population of 2,719,118 and 632,353 households. East Gojjam zone has also 21 Woreda, 480 Kebeles, 423 health posts, 102 health centers, 10 hospitals of them one is a referral hospital.

**Sample size and sampling procedure:** the study participants were selected purposively from the community until the required data reached saturation.

**Data collection procedures and instrumentation:** data was collected by Focus Group Discussions (FGDs) and in-depth interviews(IDIs) by using a semi-structured questionnaire or interview guide.

**Focused group discussion:** the interview guide was developed by reviewing different literature that addresses the objectives of the study. Before the discussion, the purpose of the study was explained by the investigators and all the discussants were agreed to discuss and allowed a tape recorder to record their discussion. Trained health professionals who had experiences in conducting FGDs had conducted/moderated the FGDs. Twelve FGDs were at the woreda health office until the data was saturated. Eight participants from the households including men, women, religious, and community leaders in the community were included in each FGD. The principal investigators participated in the selection of study participants, observation during the discussions, and transcribing tape-recorded data based on the preparation guide for FGDs. Data was collected carefully by paying attention to establish the frequency of the occurrence of themes, phrases, and expressions to make the discussants describe their opinions relative to the specific research questions.

**In-depth interview:** an IDI was conducted on sixteen HEWs that used to generate detailed information about the community´s thoughts and behavior´s regarding health extension package utilization. These interviews were used to provide context to other data offering a more complete picture of what happened in the community and why people were not utilizing health extension packages.

**Data quality control:** the questionnaire and interviewer guide was prepared separately for each data collection method as per the nature of participants was checked for its appropriateness by expert personnel. The audio file, consent form, field note, and FGD enrolment form were coded properly and consistently. Every day, every recorded audio file was checked for its appropriateness in terms of proper coding, signed consent, clear audibility, level of depth questioning, and finally consistency of the codes given for the audio file, consent, and field note.

**Data processing and analysis:** data were categorized and analyzed after the interviews were completed; the translations were done by the principal investigators manually in the field by replaying the tape recorder. The analysis was done thematically and summarized manually.

**Ethics approval and consent to participate:** ethical clearance was obtained from the research committee of Debre Marcos University and was submitted to the East Gojjam zone health department. A letter was also obtained from this department which was again submitted to each selected district health office. After permission was gained from the respected authorities, the objective of the study was explained, and written informed consent was obtained from each participant before the interview.

## Results

**Socio-demographic characteristics:** thirty-two men (4 FGDs) and sixty-four women (8 FGDs) participated in the focus group discussion from 12 woredas of East Gojjam Zone while 16 HEWs participated in the IDI from this zone. The age of the participants ranged from 24 years old to 38 years old for IDI and 27 to 76 years old for FGDs. All HEWs from IDI was grade 10 and above whereas 25 women from FGDs were illiterate. Four HEWs had six years and above work experience from IDI and 8 women and 3 men were graduated with model household training from FGD participants. Five HEWs were nurses by their background and the remaining were direct health extension workers trained from grade ten. Most of the discussants from FGD were farmers and rural dwellers. More than three fourth (76%) of the study participants were Orthodox Christian. FGD was held among households of the East Gojjam zone including women, men, and religious and community leaders on barriers of HEP utilization.

**Hygiene and sanitation:** different factors can be a barrier concerning hygiene and sanitation in East Gojjam like negligence, previous experience, misunderstand of HEP utilization, religion, unavailability of water continuously, and the environment they live in. A 52 years old, male farmer discussant said that *“I don't bother for my hygiene still now because there is no any problem that faced me due to not keeping my hygiene”*. A 46 years old woman living in a rural area describes that *“I cannot defecate and void in the toilet but I don´t know the reason why even for myself”*. The other 69-year-old male discussant who is unable to read and write living in a rural area said that *“I am experienced in storing cereals rather than the excreta, storing feces in a toilet has no value but it suffocates my compound”*. A 76 years old woman from the rural area said that *“I don´t believe that throwing waste materials anywhere that can lead to health problem rather illness is due to the GOD´s interest and it is better if I put the waste materials in my land because it fertilizes the land for better cereal production”*.

A 36 years old male governmental worker who is living in an urban area said that *"there is no continuous tap water availability in our town; sometimes we may get water after a month for different reasons”*. The other discussant from the countryside elaborate that *"we use plain water from the river, if we boil it, it may lose its nature and taste so that it is difficult to drink and may not resolve our thirst”* [27 years old man, farmer]. Concerning these issues, discussants in a group agreed that *“it is difficult that going here and there to prepare the food and we believe that the smoke itself is important for different purposes like drying meat, washed closes and serve as anti-insect agent. For such reasons we prefer cooking in the main house than separately”* [Discussant 1,4,8,17,23,34,41,55]. The other discussant from the rural area said that *“I need to follow my domestic animals´ condition throughout the night and the animals´ breath may warm my home”* [54 years old male discussant]. A discussant from a rural area said that *“it is good if it is possible to preserve the food we consume but it is meaningless to preserve the food because the environment we live in is dusty and comfortable for multiplication of germs that poison the food again"* [A 43 years´ old, rural dweller woman].

**Communicable disease prevention and control:** as we explored the barriers concerning communicable disease prevention and control in East Gojjam zone communities, the utilization is still influenced by many reasons as the discussants mentioned below. One discussant from the peasants said that *“I believe that unscreened person is healthy and I heard that HIV/AIDS is not curable so that knowing my status is meaningless rather it increases my stress”* [38 years old woman]. The other member of FGD concerning HIV/AIDS said that *“since I have no any sexual contact other than my wife it is unthinkable to be infected by HIV/AIDS because it has a good nature which is it doesn't touch anyone if she/he doesn´t touch it”* [33 years old male discussant]. The other discussant from FGD said that *“I believe that cough by itself is a self-limited and easy health problem and using home remedy is better than visiting the HEWs”* [57 years old woman]. Concerning bed net utilization, one discussant said that *“I perceive that it creates stress while I sleep under the bed net due to its bad smell and the chemical that contains”* [37 years old woman discussant].

**Family health:** respondents reported many reasons that preclude family health service utilization effectively in the East Gojjam zone as listed below: one discussant regarding family health service utilization said that *“I believe that using family planning is considered as a sin and unacceptable by our community which is forbidden for all individuals”* [31 years old, unable to read and write and rural dweller woman discussant]. The other discussant concerning family planning service said that *“I don´t use family planning forever due to its side effect like changing my wife´s behavior that leads to divorce and twin pregnancy due to GOD´s sadness and further explained that reproduction is GOD´s command which says everyone has to reproduce and reproduce to fill the earth”* [59 years old priest discussant]. One discussant regarding pregnancy and pregnancy follow-up said that *“I have no confidence in the HEWs qualification and availability of necessary resources at the HP to support me rather I want to use other alternatives like go to a private clinic or government hospitals*.” [29 years old, a government worker and urban resident woman]. The other discussant concerning breastfeeding said that *“I don´t think that my breast milk is enough for my child´s growth and my child may be thirsty if I don´t give water or cow milk for him even before six months”* [45 years old, able to read and write, farmer woman]. In-depth interviews also were taken from selected HEWs of the East Gojjam zone to explore the barriers of HEP utilization in the study area.

**Hygiene and sanitation:** one HEW about the rural community concerning latrine utilization said that *“they have no experience on latrine utilization and their children ruin the water which is prepared for hand washing after visiting the toilet. On the other hand, the nearby town waste materials are spilled out around their village negligently which indicates that the town Municipal cannot be a role model for them. Therefore, the rural community interpreted as using the toilet is not that much important”* [38 years old, 16 years´ experience HEW]. The other HEW from the town explained that *“the community in the town doesn´t use the toilet properly due to lack of water and their negligence”* [A32 years old, 7 years´ experience HEW]. There were questions raised about the reasons that preclude the community to keep personal hygiene and some rural HEWs said *“rural people are busy for farming work and many people do not bother about their hygiene due to personal negligence”* [Respondent 4, 6, and 7]. On the other side urban HEWs described *“lack of water is the main obstacle to keep personal hygiene in the community. We have tried to solve this problem by raising this issue for the hydro-power office but the office could not give a solution except giving hope to solve the problem”* [Respondent 11,2,13, and 16]. There were also assessments to observe obstacles that hindered the community to use proper and safe solid and liquid management. One rural HEW justified that *"it is their experience which we cannot reverse with education and by creating awareness about its impact. Some others thought that, “spilling waste materials everywhere especially on the farming area make it fertile*.” [>Respondent 14, age 27 with experience of 4 years]. On the other side some urban HEWs explained that *“it is the town municipal problem which could not dispose solid waste materials frequently and individuals in the town have no enough space and sewerage system to remove liquid waste materials."* [Participant 3 and 7]. Some inquiries on water supply and hygiene were done and HEWs clarified that *"children in the house haven't been given care properly which can contaminate the water. I also observe that there is not enough water supply for the community which is the country's problem as a whole”* [Respondent 7,9,10,3,4 and 11]. An investigation was done on food hygiene and almost all rural HEWs elucidated that *”the people in the community prepare food in large amount for the next day because of being busy for work in. But since they are busy, they could not get more time to eat. Then they thought eradicating foods even it is ruined is bad for the future to get edible food. Again they don´t give a concern about its hygiene even; they have a proverb “habesha´n jerm aygelewm” which shows how much they have no care about food hygiene”* [Respondent 2,7.11, and 14].

**Communicable disease prevention and control:** some questions dealt on HIV/AIDS and TB prevention and control explicated from majority rural HEWs as *“the people in the community said HIV “kalnekut aynekam” and they are fully confident as they are free from the disease. Some individuals could not visit HEWs (us) when they have persistent cough rather they use home remedies and use traditional medicine because they are afraid of quarantine from Corona Virus (COVID 19) suspicion. Again they thought it can't lead to a serious problem and they accept it as a common problem."* [Respondent 1,15,6,7]. Similarly, some justifications from almost all urban HEWs were taken as *" the people are in fear of screening for HIV which may lead stigma if they have HIV and quarantine from COVID 19 suspicion if they visit health post for a persistent cough."* [Respondent 2,5,9,10]. On the other side, there were some in-depth interviews on malaria prevention and control. There were some illustrations from rural HEWs *" since the bed net is too hard and suitable for holding materials, they are using for other purposes like collecting cereals and for making rope*." [Respondent 3,9,12,14]. Again, the rural HEWs said *"some people in the urban community have not used bed net because it is not available freely and they couldn´t afford easily to buy it."* [Respondent 3, 4 and 11].

**Family health:** there was an inquiry on family health to assess reasons which prevent youths from utilizing youth reproductive health care services in the community. Some rural HEWs said *“there is a knowledge gap where they go when they need it. Again, youths could get ashamed for using the services because sex before marriage is not socially acceptable and they couldn´t believe us since they suspect us telling somebody about their service utilization."* [Respondent 1, 2 and 9]. Similarly, one HEW from urban said that *“there is no separate service area for adolescent and youths reproductive related problem which make them not use the service."* [A 41 years old and have 14 years´ experience woman]. A similar investigation was done to assess deeply which impedes women from exclusive breastfeeding. A similar reason was found from both rural and urban HEWs, and they said *“being busy, after 3 months the children watch out for food and they don´t know the problem with giving food before 6 months.”* [Respondent age 32 with experience 4 years and age 43 with 13 years´ experience]. Some reasons hide individuals for full vaccination of their children which were explained by both urban and rural HEWs. Some said, *“side effect fear, fearing of injection pain and not believing the goal of vaccination (giving political interpretation)”* [Respondent 1,6,7,9 and 5].

**Communication and health education:** some reasons that were obstacles to health education were assessed deeply from both urban and rural HEWs. Many of them explained *“when we call them for health education, they are not cooperative with different reasons like being busy and thought no new thing will be added*.” [Respondent 1, 6, 9, and 15]. Again the other HEWs said that *"they also consider us as part of the community and don´t accept our health education, rather want strange professionals."* [Respondent 3,7,11 and 13].

## Discussion

This study was conducted to explore barriers related to health extension package utilization in the East Gojjam zone. The finding of this research revealed that many reasons influence the health extension package utilization. The majority of households in the East Gojjam zone did not utilize the HEPs properly due to negligence. This is inconsistent with the research finding done in Southern Ethiopia [12]. The other reason regarding barriers of HEP utilization which was raised by most participants is less confidence in HEWs by considering them as a part of the community rather than a health professional. This is a similar finding with studies done in the Sidama zone and overall the country, Ethiopia [13, 14]. But this is in contradiction with the Ethiopian FMOH health extension program, Health sector transformation plan, and working conditions of health extension workers in Ethiopia [1, 3, 6, 15-19].

Water is the blueprint for hygiene and sanitation thus hygiene and sanitation are unthinkable without water. Therefore, in this study shortage of water is the other main barrier related to HEP utilization especially in urban households of the East Gojjam zone. This finding is supported by a study conducted in the North Shewa zone, Ethiopia [20]. The other finding of this study is misuse and misinterpretation of the HEPs by the community typically in rural ones like using bed nets for collection of cereals and making rope rather than for malaria prevention. This is due to the strength of the bed net and its design. This finding is in agreement with the studies done in North Gondar and Southern Ethiopia [21,22] The communities´ previous experience including religious and cultural views is another reason that prevents the implementation of HEPs and it is so difficult to reverse these barriers from the community since they are considered as an unchangeable doctrine. This finding is also concurring with the studies done in China, Ghana, and Tigray, Ethiopia [23-25].

## Conclusion

The overall finding of this research revealed that many barriers undermine the utilization of health extension packages in East Gojjam Zone including religious and cultural beliefs, misuse and misinterpretation of HEPs, previous experience, negligency of the community on the implementation of HEPs, attitudes of the community towards HEWs and scarcity of water in the study area. Therefore, the government shall enforce the community to utilize the HEPs, the Zone health department should work in collaboration with the zonal water resource management office to improve water availability in urban areas of East Gojjam zone, media shall work on HEWs job description and importance of HEPs, and the HEWs strictly supervise the community in a religiously and culturally acceptable manner.

**Limitation and strength:** the strength of this study was the data source which was primary data collected directly from the study participants by FGD and IDI without any idea restriction of the respondents that makes it more accurate and helps to find the most obstacles that prevent the utilization of health extension package and the limitation of this study was the difficulty of data collection from the study participants due to COVID-19.

### What is known about this topic

- In Ethiopia, huge morbidity and mortality are due to preventable communicable diseases like malaria;
- Morbidities and mortalities related to communicable diseases are prevented through the appropriate utilization of health extension packages;
- Ethiopia had fully launched and addressed health extension packages all over the country but the utilization of the packages is low.

### What this study adds

- It identifies many barriers to health extension packages like negligence, previous experience, and misinterpretation in the study area;
- It is the base for further study at a country level at large to avoid the barriers of health extension packages.
